# The interaction of miR-181a-5p and sirtuin 1 regulated human bone marrow mesenchymal stem cells differentiation and apoptosis

**DOI:** 10.1080/21655979.2021.1915672

**Published:** 2021-04-27

**Authors:** Haitao Zhu, Hua Chen, DeGang Ding, Shui Wang, XiaoFeng Dai, YuLong Zhu

**Affiliations:** Department of Orthopedics, People’s Hospital of Sheyang County, Yancheng City, Jiangsu, China

**Keywords:** Mir-181a-5p, hBMSCs, osteoporosis, apoptosis

## Abstract

Osteoporosis (OP) characterizes a decrease in bone density and bone mass which leads to brittle fractures and serious damages to individuals. In recent years, various researches have proved that miRNAs act pivotally in the onset of bone-related diseases. In our research, we probed into the impact of miR-181a-5P on viability, differentiation, as well as apoptosis of human bone marrow mesenchymal stem cells (hBMSCs). Our study reported that overexpressing miR-181a-5p considerably reduced the cell growth, whereas the miR-181a-5p inhibition showed opposite results. Furthermore, the hBMSCs apoptosis percentage was visually elevated or minimized after overexpressing or silencing miR-181a-5p, respectively. Our data also indicated that miR-181a-5p overexpression significantly inhibited ALP activity, and level of OPN, Runx2 and OCN at mRNA and protein level, whereas miR-181a-5p inhibition presented opposite results. In addition, based on luciferase reporter assay, sirtuin 1 (Sirt1) was confirmed as the target of miR-181a-5p in hBMSCs. Finally, Sirt1 overexpression significantly inhibited the impact of miR-181a-5p mimic on apoptosis and inhibited differentiation, while silencing Sirt1 eliminated the inhibitory effects of miR-181a-5p on apoptosis and promoted differentiation via PI3K/AKT pathway. In conclusion, this work revealed that miR-181a-5p could regulate hBMSCs apoptosis as well as differentiation via regulating Sirt1/PI3K/AKT signaling pathway.

**Graphi:**
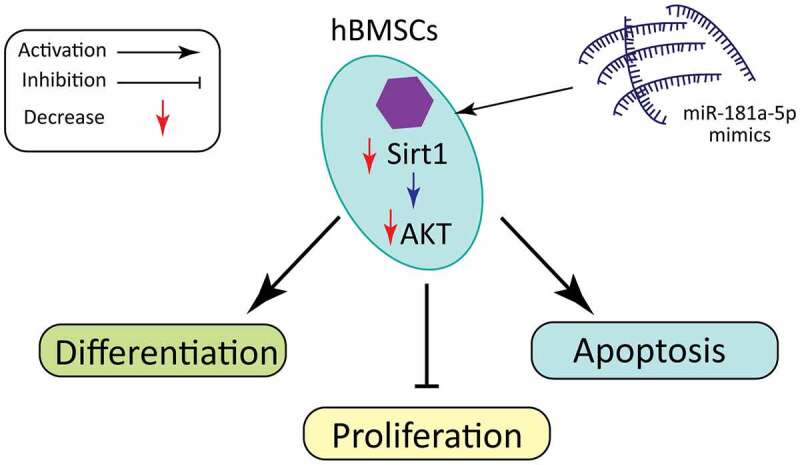
Cal Abstract

Cal Abstract

## 1. Introduction

Osteoporosis (OP), a systemic metabolic bone disease, features unbalanced relation between bone resorption and formation, internal microstructure disorder, and complete loss or decrease of strength [1–3]. In recent years, the OP incidence has gradually increased among the aged patients globally. The current treatment for OP is mainly aimed at alleviating patient discomfort symptoms, delaying the development of the disease, and reducing the risk of fragility fractures. There is still a lack of effective therapeutic targets. hBMSCs, multipotent undifferentiated progenitor cells, can differentiate into various tissue types, including cartilage, tendon, muscle cells and adipocytes. The main differentiation occurs into the osteoblasts and adipose tissue [4]. The biological characteristics of hBMSCs are constantly being studied, and their self-renewal potential and high phenotypic plasticity play an important role in regenerative medicine [5]. Therefore, elucidating the molecular pathogenesis of hBMSCs in OP is essential to treat OP.

MicroRNA (miRNA), a non-coding RNA with regulatory function, mainly binds to 3ʹUTR of target mRNA and inhibits functions of target genes [6]. MiRNAs have obvious biological functions, easy to synthesize (21–25 nucleotides), and have a good clinical applications. A number of miRNA-based therapies have entered clinical trials [7–11]. Increasing number of miRNAs is found to be closely associated with the OP progression [12]. Inoue et al. found that miR-182-PKR-IFN-β mediated osteoclast formation regulatory network and proved the therapeutic significance of miR-182 inhibition in bone protection [13]. Li et al. revealed that miR-188 is a potential therapeutic target for age-related bone loss, and primarily regulates hBMSCs osteogenesis and fat conversion [14]. Meng et al. reported that plasma miR-194-5p level is negatively correlated with bone formation in patients with osteoporosis, and inhibition of miR-194-5p can prevent osteoporosis [15]. MiRNAs are also involved in maternal influence on bone development and osteoporosis in offspring. For instance, Ellur et al. found that the high protein diet of the mothers damaged the bone mass of their descendants via miR-24-1-5p [16]. So far, several studies have revealed that miR-181-5p may act pivotally in skeletal muscle development [17]. It has also been found to target TNFα as a marker for screening those with osteoarthritis [18]. These studies suggest the role of miRNAs in OP. The use of autologous BM-MSCs for OP treatment has been widely accepted [19]. Several miRNAs also play important role in the differentiation, apoptosis and proliferation of BM-MSCs. It has been previously studied that miR-181a promotes osteoblastic differentiation through repression of TGF-β signaling molecules [20]. But the mechanism of miR-181-5p in OP is still unclear.

Therefore, our team hypothesize that miR-181-5p can regulate hBMSCs differentiation and apoptosis. Consequently, we confirmed the potential regulatory effects of miR-181a-5p on hBMSCs viability, differentiation, as well as apoptosis. In addition, we demonstrated that miR-181a-5p regulates hBMSCs differentiation as well as apoptosis through targeting Sirt1, and inhibiting PI3K/AKT pathway.

## 2. Methods

### 2.1 Cell culture

We obtained hBMSCs from ATCC, cultured them in DMEM with 10% FBS, 100 U/mL penicillin, as well as 100 g/mL streptomycin, and differentiated using β-glycerophosphate (10 mM/L), and ascorbic acid (50 g/mL, Sigma).

### 2.2 Cell viability assay

Our team seeded the hBMSCs in 96-well plates at a density of 4 × 10^4^ cells/mL for 24 hours, and transfected with miR-181a-5p mimics or inhibitors. We determined the cell viability via utilizing CCK-8.

### 2.3 Cell transfection

The hBMSCs were cultured to 70–80% confluence in a serum-free medium and transfected with the negative controls, miR-181a-5p mimics, inhibitors, si-Sirt1 or pcDNA3.1-Sirt1 after mixing with Lipofectamine 2000 in accordance with the instructions.

### 2.4 Apoptosis assay

After cell transfection for 24 hours, we centrifuged cells at 1000 g for 5 min, discarded the supernatant, collected the cells, and then gently re-suspended the cells in PBS. Then we re-suspended hBMSCs by adding 195 μL of Annexin V-FITC binding solution, 5 μL of Annexin V-FITC and 10 μL of propidium iodide staining solution, and then incubated it at 20–25°C at dark place for 10–20 minutes followed by incubation on ice. Finally, the flow cytometer was performed immediately (BD Biosciences).

### 2.5 ALP activity and ALP staining

The supernatant was obtained from the hBMSCs after cells were differentiated using 10 mM glycerophosphate, and 50 μg/mL ascorbic acid for 12 days. Blank control well, standard well and sample well were set in the 96-well plate. Added reagents in sequence according to the instructions (Beyotime Biotechnology), and then added 100 μl reaction stop solution to each well to stop the reaction. Finally, we measured the absorbance (405 nm) with a microplate reader (Thermo Fisher). Cell ALP staining was based on the instructions (Alkaline Phosphatase Staining Kit).

### 2.6 Alizarin red staining

After cell differentiation, our team washed the cells twice with FBS, fixed with 95% ethanol for 10 minutes, rinsed with distilled water for 3 times, and then incubated with 0.1% alizarin red -tris-Hcl (PH 8.3) at 37°C for 30 min. Our team acquired images by inverted microscope (Olympus Corporation, Japan).

### 2.7 Dual-luciferase reporter gene assay

Our team co-transfected Wt-Luc-Sirt1 or mut-Luc-Sirt1 with miR-181a-5p mimics/miR-181a-5p mimics NC into hBMSCs in a 12-well plate. After discarding the cell culture medium, we added 300 μL of lysis buffer to each well to lyse the cells, and determined the luciferase activity based on the instructions of dual-luciferase reporter assay kit (Beyotime Biotechnology). Renilla luciferase activity was used as internal reference.

## 2.8 qRT-PCR analysis

Our team obtained total RNA samples from hBMSCs by TRizol, using the FastKing one-step method to remove genomic cDNA and the first strand synthesis premixes (TIANGEN BIOTECH, Beijing) were used for reverse transcription using the RT specific primers. The reverse transcription (RT) primer, forward and reverse primers are given in Table 1. GAPDH and U6 were used as the experimental controls.

**Table 1.** The reverse transcription (RT) primer, forward and reverse primers

Primer sequence		
Sirt1	Forward	5ʹ-CGCCTTATCCTCTAGTTCCTGTG-3’
	Reverse	5ʹ‐TGCCTCTTGATCCCCTCCGTC‐3ʹ
Runx2	Forward	5ʹ-AAGTGCGGTGCAAACTTTCT-3’
	Reverse	5ʹ-ATGACTCTGTTGGTCTCGGTG-3’
OCN	Forward	5ʹ-TCACACTCCTCGCCCTATTG-3’
	Reverse	5ʹ-CTCTTCACTACCTCGCTGCC-3’
OPN	Forward	5ʹ-GCCGAGGTGATAGTGTGGTT-3’
	Reverse	5ʹ-AACGGGGATGGCCTTGTATG-3’
miR-181a-5p	specific primers	5ʹ-GCCGAGTAACAUUCAACGCUGU-3’
	RT primer	5ʹ-GTCGTATCCAGTGCAGGGTCCGAGGTATT CGCACTGGATACGACACTCACCG-3ʹ
U6	specific primers	5ʹ-TGCGGGTGCTCGCTTCGGCAGC-3’
	RT primer	5ʹ-GTCGTATCCAGTGCGTGTCGTGGAGTCGGCA ATTGCACTGGATACGACAAAATATGGAAC-3’
	Reverse	5ʹ-AACGGGGATGGCCTTGTATG-3’

### 2.9 Western Blot

We obtained the total proteins from hBMSCs using RIPA Lysis Buffer (Beyotime Biotechnology) with 1 mM phenylmethyl sulfonyl fluoride. Our team applied the Enhanced BCA Protein Assay Kit to determine Protein concentrations; separated protein samples via SDS-PAGE (10–12%); then transferred to PVDF. We used the proteins on the PVDF membrane as antigens to bind to the corresponding antibodies, and then reacted with the isotope-labeled second antibodies. Finally, the proteins exposed by specific target antibodies were detected by chemiluminescence substrates.

### 2.10 Human clinical samples

During the period from March 2017 to November 2018, bone tissue specimens were collected from 10 patients with OP and 10 normal subjects at the Department of Orthopedic Surgery of our hospital after proper approval from Ethical Committee in accordance with the Helsinki Declaration. Written informed consents were obtained from the patients or their guardians.

### 2.11 Statistical Analysis

We analyzed all data via GraphPad Prism 8.1, expressed all values as mean ± SD, and applied Student’s t test for statistical analysis. *P < 0.05 and ***P < 0.01 means statistical significance.

## 3. Results

### 3.1 MiR-181a-5p inhibits hBMSCs viability

**Figure 1.**
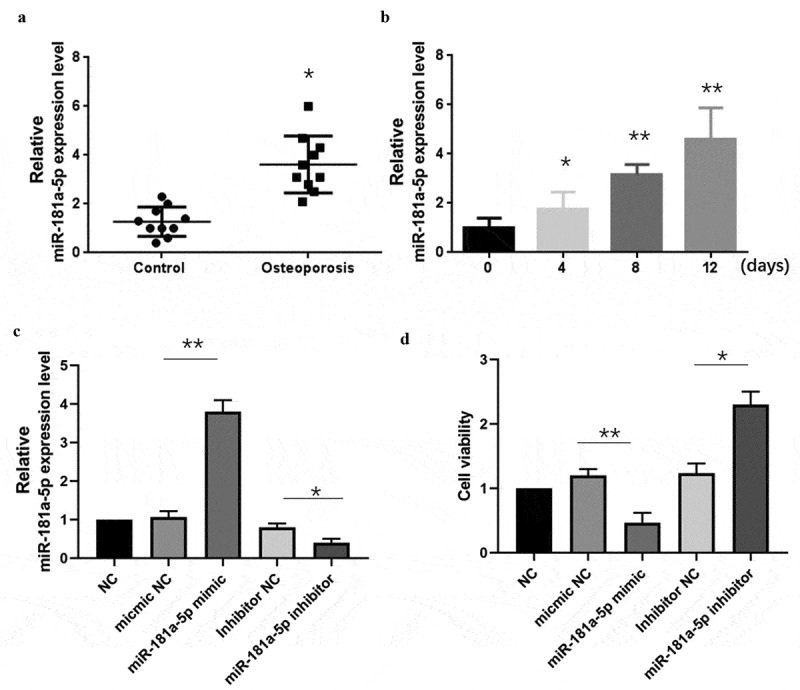
Effect of miR-181a-5p on the hBMSCs viability.A. Expression of miR-181a-5p on OP specimens. B. Expression of miR-181a-5p in β-glycerophosphate induced hBMSCs cell at different time intervals. C. The expression of miR-181a-5p in hBMSCs of different treatment groups was determined by RT-qPCR. **D**. The effect of overexpression or inhibition of miR-181a-5p on the hBMSCs viability was detected by CCK-8 experiment. n = 3, *p < 0.05 and **p < 0.01

It has been studied that miR-181a promotes osteoblastic differentiation. However, the exact role of miR-181a-5p in hBMSCs is not well documented. Therefore, we designed this study to evaluate the role of miR-181a-5p in the hBMSCs cell differentiation and apoptosis. Firstly, we used human OP specimens and used RT-qPCR to evaluate the expression of miR-181a-5p. Results showed that miR-181a-5p was significantly increased in OP specimens as compared to the control (Figure 1a). Furthermore, we observed miR-181a-5p expression in the hBMSCs after transfection with β-glycerophosphate, an important mediator to transdifferentiate hBMSCs, for different days. These results showed that as the time pass for the differentiation of hBMSCs, the expression of miR-181a-5p increases (Figure 1b). Next, we transfected the hBMSCs cells with miR-181a-5p mimic and miR-181a-5p inhibitor to modulate the miR-181a-5p expression. We found that miR-181a-5p mimic and inhibitor up-regulated and down-regulated the miR-181a-5p expression, respectively (Figure 1c). MiR-181a-5p levels did not significantly change in mimic NC group and inhibitor NC group. MiR-181a-5p mimics & inhibitors inhibited or promoted hBMSCs viability, respectively (Figure 1d). However, mimics & inhibitors NC did not affect cell viability.

### 3.2 MiR-181a-5p promoted hBMSCs apoptosis

**Figure 2.**
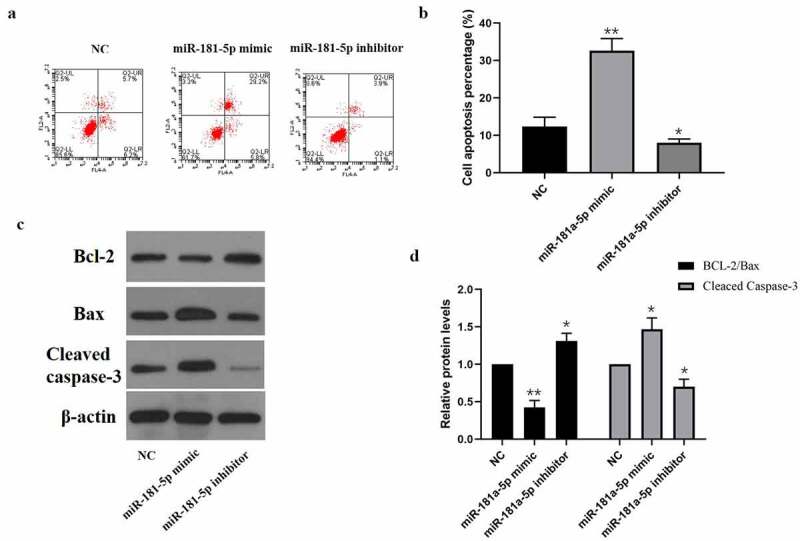
Effect of miR-181a-5p on the hBMSCs apoptosis. **A. B**. Apoptosis of hBMSCs in different treatment groups was detected by flow cytometry. **C. D**. Expression of apoptosis marker proteins (Cleaved caspase3, Bcl2, and Bax) in different treatment groups. n = 3, *p < 0.05 and **p < 0.01

Next, we determined the impact of miR-181a-5p on hBMSCs apoptosis via flow cytometry. As shown in Figure 2a and 2b, miR-181a-5p mimics & inhibitors promoted or inhibited hBMSCs apoptosis percentage, respectively. As shown in Figure 2c and 2d western blotting results, miR-181a-5p overexpression considerably down-regulated the Bcl2/Bax ratio and up-regulated Cleaved caspase3 expression, whereas inhibition of miR-181a-5p visually up-regulated the Bcl2/Bax ratio and down-regulated Cleaved caspase-3 expression. These data suggested that overexpression of miR-181a-5p and inhibition of miR-181a-5p promoted and inhibited cell apoptosis, respectively.

### 3.3 Inhibition of miR-181a-5p promoted hBMSCs differentiation

**Figure 3.**
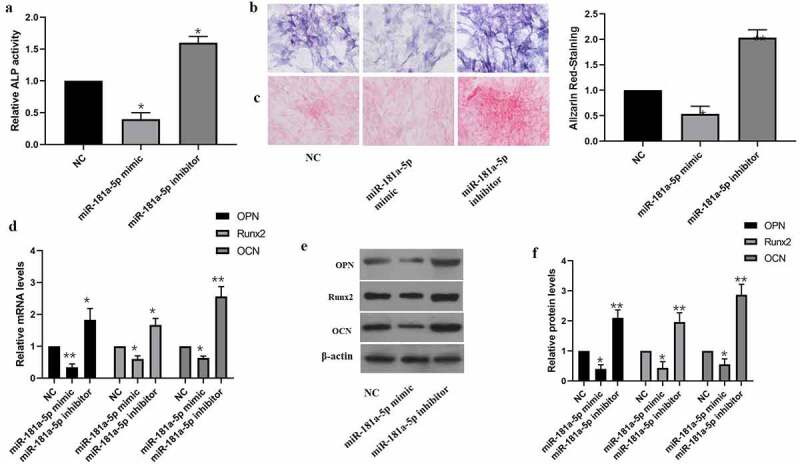
Effect of miR-181a-5p on the hBMSCs differentiation. **A**. ALP activity was detected by the ALP activity kit. **B**. ALP staining. **C**. Alizarin red staining. **D**. The expressions of OPN, OCN and Runx2 mRNA were detected by qRT-PCR. **E. F**. The expressions of OPN, OCN and Runx2 protein were detected by western blot. n = 3, *p < 0.05 and **p < 0.01

Our team tested the impact of miR-181a-5p on hBMSCs differentiation as well. Inhibition of miR-181a-5p elevated the ALP activity as well as ALP staining intensity (Figure 3a & 3b). Furthermore, inhibition of miR-181a-5p elevated alizarin red staining intensity (Figure 3c). The findings of RT-qPCR together with western blotting supported that miR-181a-5p overexpression inhibits OPN, OCN and Runx2 at mRNA and protein level, while inhibition of miR-181a-5p increased level of OPN, OCN and Runx2 (Figure 3d-f). In brief, miR-181a-5p inhibited hBMSCs differentiation.

### 3.4 MiR-181a-5p targeted Sirt1 in hBMSCs

**Figure 4.**
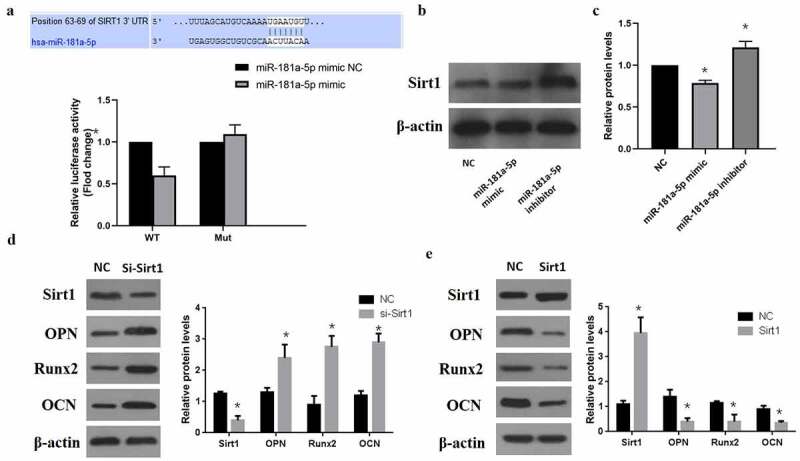
MiR-181a-5p targeting Sirt1. **A**. Dual Luciferase Reporter assay showed miR-181a-5p targeting Sirt1. **B. C**. Protein expression level of Sirt1 after overexpression or silencing of miR-181a-5p. **D**. Protein expression of transdifferentiation genes after sirt1 silencing. **E**. Protein expression of transdifferentiation genes after sirt1 overexpression. n = 3, *p < 0.05 and **p < 0.01

MiRNAs bind with the 3ʹUTR of target mRNA and inhibit their expression. In our study, we found that miR-181a-5p targets the Sirt1 3ʹUTR (Figure 4a). According to the luciferase report results, miR-181a-5p overexpression can minimize Wt-luciferase activity, whereas miR-181a-5p overexpression doesn’t affect the luciferase activity in mutant type (Figure 4a). Moreover, we further verified that in comparison with NC group, miR-181a-5p overexpression observably decreased Sirt1 expression, while inhibition of miR-181a-5p could elevate Sirt1 expression (Figure 4b & 4c). Therefore, we concluded that Sirt1 is a target of miR-181a-5p. Then we performed western blot analysis and determined the effects of sirt1 in the differentiation of hBMSCs. Our results showed that silencing of sirt1 significantly increased the hBMSCs differentiation markers OPN, Runx2, and OCN (Figure 4d). However, overexpression of sirt1 significantly decreased these transdifferentiation markers (Figure 4e). These results suggested that sirt1 takes part in the differentiation of hBMSCs, whereas miR-181a-5p could target the 3ʹUTR to alter the role of sirt1 in hBMSCs.

### 3.5 MiR-181a-5p induced hBMSCs apoptosis and differentiation by inhibiting Sirt1

**Figure 5.**
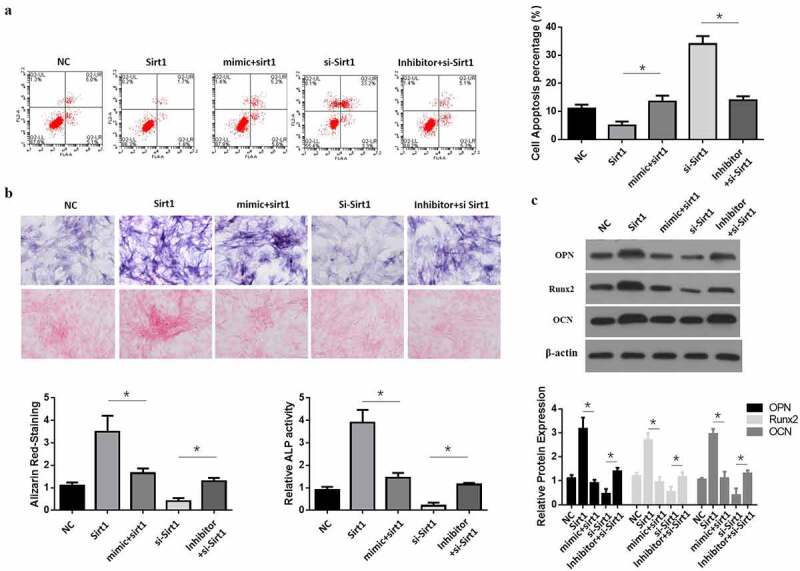
MiR-181a-5p regulate the differentiation and apoptosis of hBMSCs through Sirt1. **A. B**. After overexpression and silence of miR-181a-5p or overexpression and silence or over expression of Sirt1, the apoptosis of hBMSCs was detected by flow cytometry (a), and hBMSCs were stained with Alizarin Red and ALP activity (b). **C**. Protein expression of transdifferentiation genes after overexpression and silence of miR-181a-5p or overexpression and silence or over expression of Sirt1. n = 3, *p < 0.05 and **p < 0.01

Sirt1 overexpression reduced hBMSCs apoptosis; while Sirt1 and miR-181a-5p overexpression at the same time induced hBMSCs apoptosis (Figure 5a). In comparison with the sirt1 inhibition group, simultaneous inhibition of Sirt1 with inhibition of miR-181a-5p decreased cell apoptosis. Sirt1181a-5p overexpression induced hBMSCs differentiation, while Sirt1 and miR-181a-5p overexpression at the same time reduced the hBMSCs differentiation and ALP activity (Figure 5b), while in comparison with the miR-181a-5p suppression group, inhibition of Sirt1 and miR-181a-5p at the same time increased cell differentiation (Figure 5d). These results suggested that miR-181a-5p induced hBMSCs apoptosis and differentiation by inhibiting Sirt1.

**3.6 MiR-181a-5p alters the PI3K/Akt signaling pathway through Sirt1 to promote apoptosis and differentiation of hBMSCs**

**Figure 6.**
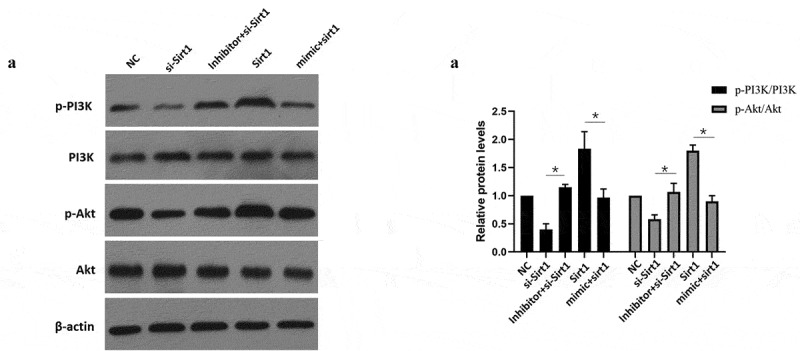
MiR-181a-5p regulated the PI3K/AKT pathway via Sirt1 in hBMSCs. **A. B**. After overexpression and silence of miR-181a-5p or overexpression and silence of Sirt1, the PI3K/AKT of hBMSCs was detected by western blot. n = 3, *p < 0.05 and **p < 0.01

Sirt1 overexpression increased p-PI3K/PI3K as well as p-Akt/Akt ratios, but Sirt1 overexpression together with miR-181a-5p overexpression reduced p-PI3K/PI3K and p-Akt/Akt ratios (Figure 6a-b). Inhibition of sirt1 reduced activation of p-PI3K and p-Akt in comparison with NC group, while simultaneous inhibition of Sirt1 and miR-181a-5p induced the activation of p-PI3K and p-Akt (Figure 6a-b). Based on above data, we concluded that miR-181a-5p could alter PI3K/Akt signaling pathway through Sirt1 to promote apoptosis and differentiation of hBMSCs.

## 4. Discussion

MiRNA, a kind of non-coding and single-stranded RNA molecule, consists of 21–25 nucleotides binds to the complementary sequence of mRNA in 3ʹUTR region to block protein translation and regulate mRNA stability [3,21]. Since the discovery of miRNA, it has been found that miRNA acted pivotally in regulating various diseases [10,22]. Previous studies have found that about 30% of protein-coding genes in mammals are controlled by miRNAs, which participates in regulation of almost every cellular process that has been studied [23]. hBMSCs can differentiate into osteoblasts and chondroblasts, and acts pivotally in osteoporotic diseases [24]. In our research, our team confirmed that miR-181a-5p significantly inhibited hBMSCs viability. Therefore, our members hypothesized that miR-181a-5p could alleviate osteoporosis through regulating hBMSCs differentiation and apoptosis.

Further studies have found that overexpression of miR-181a-5p significantly promoted hBMSCs apoptosis. At present, there are many studies have concluded the effects of drugs or other factors on hBMSCs apoptosis and its pro-apoptotic mechanism. Among them, it is found that apoptosis of hBMSCs is accompanied by a rise in pro-apoptotic proteins Cleaved caspase-3 as well as Bax levels, and a drop in anti-apoptotic proteins Bcl-2 levels [25–28]. Based on our work, miR-181a-5p visually stimulated cleaved caspase3 expression and inhibited the Bcl2/Bax ratio. In summary, our experiment supported that miR-181a-5p might be a promoter of hBMSCs apoptosis.

A derivative (Anthraquinone) of the Anthraquinone family, alizarin forms a compound with calcium salts to identify the calcium salts in tissues and cells. Change in calcium salt is one of the markers for bone cell proliferation and differentiation, and bone tissue osteogenic potential. Staining with alizarin produces orange-red deposits [29]. Alkaline phosphatase is widely distributed in human bones, intestines, kidneys, liver, placenta and other tissues, with the liver the most. It is a commonly used clinical screening and diagnosis index for hepatobiliary diseases and bone diseases [30]. Runx2 regulates the differentiation of hBMSCs into osteoblasts and chondrocytes, and promotes the transcription and expression of OCN, OPN, and BSP proteins [31,32]. In this research, our team revealed that miR-181a-5p overexpression visually reduced ALP activity as well as Alizarin Red staining density, and also reduced OPN, OCN as well as RUNX2 protein expressions. But silencing of miR-181a-5p significantly increased these above mentioned indicators. These findings indicate that miR-181a-5p might inhibit hBMSCs differentiation into osteoblasts.

SIRT1, a type 3 histone deacetylase, is dependent on nicotinamide adenine dinucleotide as well as an essential factor in regulating biological life span [33]. According to recent studies, SIRT1 acts pivotally in the physiological activities of bone tissue [34]. In vitro research has revealed that SIRT1 can promote osteogenic hBMSCs differentiation as well as inhibit osteoclast-mediated bone resorption. SIRT1 knockout mice have a low bone mass phenotype, but specific signaling pathway is not clear in the in vivo experiments [35]. We have demonstrated that Sirt1 is a target of miR-181a-5p. MiR-181a-5p overexpression inhibited Sirt1 expression in hBMSCs. Compared with the overexpression of miR-181a-5p, the simultaneous overexpression miR-181a-5p and Sirt1 group significantly promoted the differentiation of hBMSCs, and inhibited hBMSCs apoptosis. These findings supported that miR-181a-5p regulate differentiation and apoptosis of hBMSCs through Sirt1. Recent studies have found that the PIK/AKT pathway acts pivotally in the osteoblast’s differentiation and apoptosis [36,37]. But whether Sirt1 regulates hBMSCs differentiation and apoptosis through the PI3K/AKT pathway is not clear. This is the first study to report that miR-181a-5p overexpression is important to inhibit PI3K/AKT pathway, while Sirt1 overexpression alleviates the inhibition of PI3K/AKT via miR-181a-5p. To sum up, miR-181a-5p/Sirt1 axis is available to regulate hBMSCs differentiation and apoptosis through the PI3K/Akt pathway.

Overall, this study supports that miR-181a-5p may partially regulate hBMSCs differentiation and apoptosis through regulating Sirt1 and Sirt1/AKT axis. The impact of miR-181a-5p on hBMSCs together with osteoblasts still need further studied to explain in detail how miR-181a-5p serves in osteoporosis.

## Data Availability

The data set applied and/or analyzed amidst existing studies are accessible to corresponding author on rational request.

## Highlights

1. **MiR-181a-5p promoted hBMSCs apoptosis and inhibited the cell viability.**
2. **Inhibition of miR-181a-5p is associated with the increased hBMSCs differentiation.**
3. **MiR-181a-5p play vital** inhibitory **role in the Sirt1/PI3K/Akt signaling pathway to promote apoptosis and differentiation of hBMSCs**
